# A major quantitative trait locus for wheat total root length associated with precipitation distribution

**DOI:** 10.3389/fpls.2022.995183

**Published:** 2022-08-24

**Authors:** Huangxin Chen, Jiatai Wei, Rong Tian, Zhaoyong Zeng, Huaping Tang, Yanlin Liu, Qiang Xu, Mei Deng, Qiantao Jiang, Guoyue Chen, Yaxi Liu, Wei Li, Pengfei Qi, Yunfeng Jiang, Yun Jiang, Liwei Tang, Yuming Wei, Youliang Zheng, Xiujin Lan, Jian Ma

**Affiliations:** ^1^State Key Laboratory of Crop Gene Exploration and Utilization in Southwest China, Sichuan Agricultural University, Chengdu, China; ^2^Triticeae Research Institute, Sichuan Agricultural University, Chengdu, China; ^3^College of Agronomy, Sichuan Agricultural University, Chengdu, China; ^4^Institute of Biotechnology and Nuclear Technology Research, Sichuan Academy of Agricultural Sciences, Chengdu, China; ^5^Panzhihua Academy of Agricultural and Forestry Sciences, Panzhihua, China

**Keywords:** total root length, quantitative trait loci, wheat, Wheat55K SNP array, parental reproductive environment, precipitation

## Abstract

Optimizing root system architecture (RSA) allows crops to better capture water and nutrients and adapt to harsh environment. Parental reproductive environment (PRE) has been reported to significantly affect growth and development throughout the life cycle of the next generation. In this study, 10 RSA-related traits were evaluated in seedling stage from five independent hydroponic tests using seeds harvested from five different PREs. Based on the Wheat55K SNP array-based genetic map, quantitative trait loci (QTL) for these traits were detected in a recombinant inbred line population. Twenty-eight putative QTL for RSA-related traits were detected, covering thirteen chromosomal regions. A major QTL, *QTrl.sicau-2SY-4D* for total root length (TRL), which was likely independent of PREs, explained 15.81–38.48% of phenotypic variations and was located at 14.96–19.59 Mb on chromosome arm 4DS. Interestingly, it showed pleiotropic effects on TRL, root area, root volume, root forks, root dry weight, and shoot dry weight. The functional marker *KASP-Rht-D1* for *Rht-D1* was used to genotype 2SY population and remapping QTL for TRL showed that *QTrl.sicau-2SY-4D* was not linked to *Rht-D1.* The kompetitive allele-specific PCR (KASP) marker, *KASP-AX-110527441* linked to this major QTL, was developed and used to successfully validate its effect in three different genetic populations. Further analysis suggested that the positive allele at *QTrl.sicau-2SY-4D* was mainly utilized in wheat breeding of northwest China where precipitation was significantly lower, indicating that wheat requires longer TRL to capture water and nutrients in arid or semi-arid regions due to deficient precipitation. Additionally, four genes (*TraesCS4D03G0059800*, *TraesCS4D03G0057800*, *TraesCS4D03G0064000*, and *TraesCS4D03G0064400*) possibly related to root development were predicted in physical interval of *QTrl.sicau-2SY-4D*. Taken together, these results enrich our understanding on the genetic basis of RSA and provide a potentially valuable TRL QTL for wheat breeding.

## Introduction

Wheat is counted among the “Big three” cereal crops because of its better environmental adaptability, richer nutrients, and higher yield potential (Shewry, 2009). Plant breeders constantly perform genetic improvement of yield to meet the needs of human beings (Cao et al., 2020). For instance, the successful utilization of *Rht-B1* and *Rht-D1* led to a significant increase in wheat productivity (Peng et al., 1999). Both root and shoot are vital components for wheat morphology, whereas the genetic improvement of root is far behind shoot (Waines and Ehdaie, 2007). Root system architecture (RSA) represents the shape and spatial distribution of roots underground, including root length, diameter, number, angle, density and so on (Li et al., 2021). RSA is involved in water and nutrients utilization, production and storage of organic matter, bracing and anchoring, resisting abiotic stress, and interaction with soil microenvironment (Maqbool et al., 2022). Breeding wheat varieties with optimized RSA is expected to increase world grain yield.

Numerous studies in RSA-associated genes or quantitative trait loci (QTL) have been identified in model species. For rice, *DEEPER ROOTING 1* and its homolog *quantitative trait loci for SOIL SURFACE ROOTING 1* were negatively regulated by auxin and participate in the gravity responses of roots (Uga et al., 2013; Kitomi et al., 2020). *OsABA8ox*, encoding an abscisic acid 8′-hydroxylase, suppressed root elongation of seedling (Zhang et al., 2020). *SR1* (*SHORT-ROOT 1*) contained an EXO70 domain and its mutation impacted the whole development process of root (Xing et al., 2021). For *Arabidopsis*, loss-of-function mutations in *MEDIATOR18* inhibited growth of primary root and promoted development of lateral root and root hair (Raya-González et al., 2018). *LATERAL ORGAN BOUNDARIES DOMAIN* inhibited cytokinin signaling to promote secondary growth of root (Ye et al., 2021). *PAMP-INDUCED SECRETED PEPTIDE 2* was an auxin response gene and affected root elongation by controlling cell division and elongation (Hussain et al., 2021).

Compared with rice and *Arabidopsis*, the study about RSA-related traits in wheat is extremely hindered because of the huge and complex hexaploid genome (Dong et al., 2015). However, a few genes have been revealed recently. *LATERAL ROOT DENSITY*, a *KNAT3* ortholog of *Arabidopsis*, negatively regulated root development under water stress (Placido et al., 2020). *TaVSR1-B* encoded a vacuolar sorting receptor protein and influenced root length by regulating the ratio of elongation region to differentiated zone at the booting stage (Wang J. et al., 2021). *ENHANCED GRAVITROPISM 2* encoded a sterile alpha motif domain-containing protein and played a pivotal role in regulating root gravitropism (Kirschner Gwendolyn et al., 2021).

With the development of molecular technologies, there have been many reports about QTL of RSA-related traits in wheat over the past decade (Liu et al., 2013; Merchuk-Ovnat et al., 2017; Alemu et al., 2021). For example, thirty-three QTL were detected for RSA-related traits by 35K DArT array and 17K SNP array (Zheng et al., 2019). Twelve major chromosomal regions associated with RSA were identified through genome-wide association analysis (Xu et al., 2021). A total of 158 stable QTL for 27 morphological and physiological traits of root and shoot were evaluated based on Wheat55K SNP array in a recombinant inbred line (RIL), and 7 QTL intervals were validated in other genetic backgrounds (Luo et al., 2021). Although numerous QTL for RSA were identified, few of them were major, stably expressed and validated, resulting in that they were hard to be utilized in breeding.

Additionally, it was reported that life history environment of the parental generation can affect growth and development throughout the life cycle of the next generation to an extent that should be considered when performing genetic studies in plants (Elwell et al., 2011). For example, drought initiation of parents in wheat enhanced the tolerance of progenies to drought due to the accumulation of proline and glycine betaine (Wang et al., 2018). Offspring of the stressed maternal generation can maintain longer roots under low biomass allocation at drought in barley (Nosalewicz et al., 2016). Progenies with warm parental reproductive environment (PRE) had faster germination rates and root elongation growth at different temperatures than those with cold PRE in *Arabidopsis* (Blödner et al., 2007). Besides, PRE affected the size of the progeny seed in *Arabidopsis*, which was closely associated with root growth and geotropism (Elwell et al., 2011). These previous studies suggested that it is essential to identify QTL associated with RSA using seeds harvested from different environments (i.e., PREs) aiming at detecting stably expressed loci that were independent of PREs as suggested by Elwell et al. (2011) given that they may have more breeding potential. However, to our knowledge, very few of previous studies have considered the influence of PREs on the authenticity, reliability, and stability of identified QTL related to RSA.

In this study, we conducted five independent hydroponic tests using seeds harvested five different environments, respectively, at seedling stage to identify major and stable QTL for ten RSA-related traits based on the previous constructed high-density Wheat55K SNP array. The genetic relationships among RSA-related traits and agronomy traits were analyzed. The effects of major QTL in multiple genetic backgrounds were validated. Geographic distribution and frequency of the alleles of the presently identified major QTL were further analyzed in Chinese ten agro-ecological zones. Additionally, candidate genes of the major QTL were predicted. This study can provide key information for the optimization of RSA in wheat breeding.

## Materials and methods

### Plant materials

One mapping population (20828/SY95-71, 2SY, 126 F_7_ RILs) and two validation populations (HTG3/SY95-71, HTG3SY, 131 F_3_ lines; MZ5/SY95-71, MZ5SY, 170 F_4_ lines) were used to study RSA-related traits. 20828, SY95-71, HTGW3, and MZ5-2 are advanced lines that have been utilized in wheat breeding. They were stored in seed repository of Triticeae Research Institute in Sichuan Agricultural University. Besides, a panel containing 717 Chinese wheat landraces (CWL) from ten agro-ecological zones in China (Zhou et al., 2018) was further used to study the geographic distribution and effects of positive allele at the major QTL.

### Phenotypic analysis

The 2SY population was grown in five different environments including Ya’an (103°0′ E, 29°58′ N) in 2017 and 2018 (T1 and T2, T for test), Chongzhou (103°38′ E, 30°32′ N) in 2018 and 2019 (T3 and T4), Wenjiang (103°51′ E, 30°43′ N) in 2019 (T5) of Sichuan Province in China as described in a previous study (Qu et al., 2021). The HTG3SY and MZ5SY populations were grown in Chongzhou in 2021. Planting and field management strategy were conducted following the procedures described by Liu et al. (2020). We harvested their seeds and then evaluated the phenotype of root at seedling stage using a hydroponic culture method. The seeds used in each test (T1–T5) had completely different PREs. The experiment was repeated three times with a completely random design.

Hydroponic culture method was proceeded in a greenhouse as described in previous study (Ma et al., 2017). Twenty plump and similar-sized seeds were selected from each line of investigated populations. The seeds were sterilized with 10% sodium hypochlorite for 5 minutes, and washed with distilled water and placed in a box covered by filter paper. After a week, three seedlings with similarly healthy growth were removed the endosperm and moved to a plastic box (50 cm × 40 cm × 30 cm) containing Hoagland nutrient solution (replaced weekly). Oxygen was pumped to Hoagland nutrient solution using air pump. In order to minimize the experimental errors, the seedlings of all lines in each test were sprouted, grown, and measured simultaneously. Root and shoot tissues were collected after four weeks. Epson Expression 10000 XL^1^ was utilized for root image obtaining. Win-RHIZO 2013e 32bit^2^ was used to analyze the total root length (TRL, cm/plant), root area (RA, mm^2^/plant), root volume (RV, mm^3^/plant), root diameter (RD, mm/plant), root tips (RT, count/plant), and root forks (RF, count/plant). The RDW (g/plant) and shoot dry weight (SDW, g/plant) were measured using an electronic balance after root and shoot tissues dried to a constant weight at 105 °C. The dry root–shoot ratio (DRS, RDW/SDW) was calculated. The root number (RN, count/plant) was estimated from the root image.

Additionally, the best linear unbiased prediction (BLUP) datasets of the plant height (PH) (Liu et al., 2020), spike extension length (SEL) (Li C. et al., 2020), tiller number (TN) (Liu et al., 2020), spikelet number per spike (SNS) (Ding et al., 2021), and thousand-grain weight (TGW) (Qu et al., 2021) in 2SY population were derived from our previous studies. BLUP dataset of the TRL in CWL was provided by Lin et al. (2020), and that of the TN, SNS, and TGW in CWL were provided by Liu et al. (2017). The annual precipitation data during 2000–2015 in China was collected from the Resource and Environment Science and Data Center, Institute of Geographic Sciences and Natural Resources Research.^3^ It was used to speculate the potential relationship between the geographic distribution of positive allele for *QTrl.sicau-2SY-4D* and regions with different precipitation.

### Statistical analysis

The mean values of phenotypic data from three repeated tests were used for subsequent analysis. The BLUP and broad hereditary capacity (*H*^2^) of each corresponding trait were evaluated via SAS v8.0.^4^ *H*^2^ was estimated as follow: $H^{2}=\sigma_{G}^{2}/\left(\sigma_{G}^{2}+\sigma_{e}^{2}r\right)$, with $\sigma_{G}^{2}$ = genetic variance, $\sigma_{e}^{2}$ = residual variance, and *r* = number of replicates. IBM SPSS Statistic v27^5^ was used to perform Student’s *t*-test (*P* < 0.05) and Pearson’s correlation analysis. OriginPro 2021 v9.8^6^ was used to draw frequency distribution, correlation plot, and boxplot. ArcGIS Desktop v10.5^7^ was used for processing of the data for mean annual precipitation in China.

### Quantitative trait loci analysis

A previously published high-density genetic linkage map constructed by the Wheat55K array was used in this study (Liu et al., 2020). It contained 9,030 polymorphic markers, with a total length of 4273.03 cM and a mean marker densities of 1.69 cM/marker.

QTL mapping was performed by the inclusive composite interval mapping-additive and dominance (ICIM-ADD) of biparental population (BIP) module using IciMapping v4.2 (Meng et al., 2015). The walking speed of all QTL was set at 1.0 cM with the PIN value of 0.001. The logarithm of odds (LOD) threshold was set as 3.51 (TRL, RA, RV, and RF), 3.62 (RD), 3.48 (RT), 3.52 (RDW and RN), 3.59 (SDW), and 3.28 (DRS) by 1000 permutation tests with the type I error of 0.05. Only QTL that was detected at least in two tests and which phenotype variance explained (PVE) was greater than 10% was considered as stable and major one. QTL were named according to the International Rules of Genetic Nomenclature (‘sicau’ means Sichuan Agricultural University) (McIntosh et al., 2013). The graphical representations of QTL on linkage groups were drawn using MapChart v2.3.2.^8^

Conditional QTL analysis was applied to study the genetic relationship between complex traits and their components at single QTL level (Cui et al., 2011). We investigated the genetic relationships between TRL and other RSA-related traits, PH, and SEL by Conditional QTL. The conditional phenotype values (Trait 1| Trait 2) were calculated using QGAStation v2.0 (Chen et al., 2012), where Trait 1| Trait 2 indicated that value of Trait 1 was conditioned on Trait 2. The conditional QTL and its LOD and additive effect values were calculated using IciMapping 4.2. The walking speed, PIN value, and LOD threshold were set at 1.0 cM, 0.001, and 3, respectively. By comparing the LOD and additive effect values of conditional QTL and unconditional QTL, the influence of other traits on target QTL could be evaluated (Cui et al., 2011; Fan et al., 2019). For example, the LOD and additive effect values were changed significantly but was greater than 3 indicating that the QTL was partially affected by other traits, reduced to less than 3 meaning that the QTL was completely affected by other traits, and were no difference suggesting that the target QTL was not completely affected by other traits.

### Marker development and quantitative trait loci effects analysis

To further analyze the effects of identified major QTL, we developed a kompetitive allele-specific PCR (KASP) marker from the closely linked flanking marker of the major QTL, and genotyped the segregating lines in different genetic backgrounds (HTG3SY and MZ5SY) followed the method of Li C. et al. (2020). The flanking marker, *AX-110527441*, was converted into KASP marker *KASP-AX-110527441* (Supplementary Table 1). CFX96*™* Real-Time System was used for genotyping. The whole volume of 10 μL containing 0.75 μL template DNA, 1.4 μL mixture of forward and reverse primers, 2.85 μL deionized water, and 5 μL SsoFast EvaGreen mixture was used for the amplification reactions. PCR reaction procedure: 15 min at 94°C, 40 cycles of 20 s at 94°C, and 60 s at 61–55°C with per cycle dropping 6°C.

The genotypes of HTG3SY and MZ5SY populations were screened respectively into three categories based on *KASP-AX-110527441*: lines with the homozygous alleles from HTGW3 or MZ-5, homozygous ones from SY95-71, and heterozygous ones, respectively. Zhou et al. (2018) genotyped the 237 lines of CWL using *AX-110527441* based on Wheat660K SNP array, and we further used the *KASP-AX-110527441* to genotype the remaining lines. CWL were also divided into three categories: lines with the homozygous alleles same as SY95-71, homozygous ones different from SY95-71, and heterozygous ones, respectively. Finally, phenotypic differences between the lines of two categories with homozygous alleles were analyzed by Student’s *t*-test (*P* < 0.05).

In addition, the functional KASP marker (*KASP-Rht-D1*, Supplementary Table 1) for *Rht-D1* designed by Rasheed et al. (2016) was used to preliminarily analyze the relationship between the major QTL of our study and *RhtD1*. The genotypes of 2SY population was screened based on *KASP-Rht-D1* and the genetic map of the 4D chromosome was reintegrated by JoinMap v4.0 (Van Ooijen, 2006).

### Comparison with reported quantitative trait loci and prediction of candidate genes

The sequences of the flanking markers linked with the major QTL in this study were derived from our previous study (Liu et al., 2020), and sequences of flanking markers for reported QTL were downloaded from T3/Wheat^9^ and GrainGenes 3.0.^10^ Their physical intervals were compared by blasting Their physical intervals were compared against (*E*-value of 1e-5) the genome assemblies of *Triticum aestivum* cv. Chinese Spring (CS v2.1 genome) (Zhu et al., 2021) on WheatOmics (Ma et al., 2021). The annotations and the expression patterns (International Wheat Genome Sequencing Consortium [IWGSC] et al., 2014) for predicted genes were also searched on WheatOmics. Further the common predicated genes between flanking markers were retrieved from protein sequence by comparing CS v2.1 genome and Aet v5.0 genome (*Aegilops tauschii* acc. AL8/78.) (Wang L. et al., 2021).

## Result

### Phenotypic performance

**FIGURE 1:**
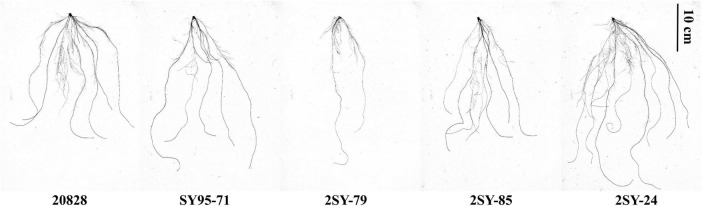
Root morphology for the parents and partial lines.

**FIGURE 2:**
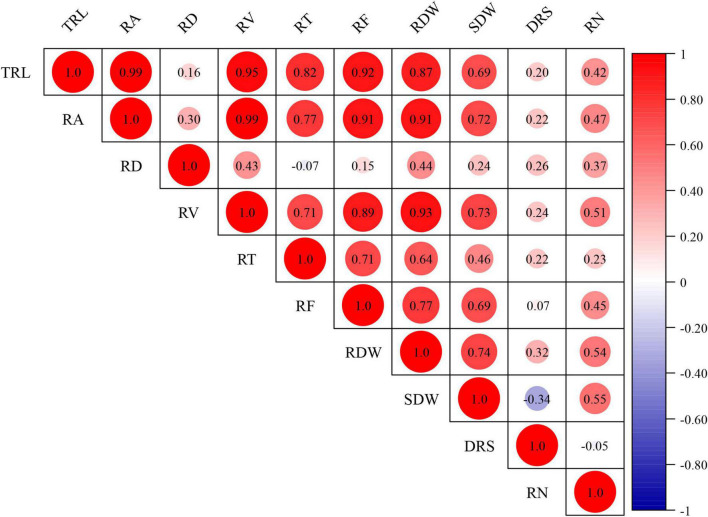
Correlation coefficients for RSA-related traits. *TRL* total root length, *RA* root area, *RV* root volume, *RD* root diameter, *RT* root tips, *RF* root forks, *RDW* root dry weight, *SDW* shoot dry weight, *DRS* dry root–shoot ratio, *RN* root number. The BLUP datasets were used to evaluate the correlation coefficients for RSA-related traits.

Parents of 2SY population and their RILs exhibited significant differences for ten RSA-related traits in five tests (Figure 1 and Table 1). The TRL, RA, RT, RF, and RN of 20828 were significantly higher (*P* < 0.05) than those of SY95-71 in at least two tests, whereas the SDW was significantly lower (*P* < 0.05). There were small phenotypic differences for RV, RD, RDW, and DRS between parents. The RSA-related traits of the 2SY RILs ranged from 188.19 to 1931.78 cm for TRL, 21.19 to 195.94 mm^2^ for RA, 0.27–0.47 mm for RD, 0.19–1.95 mm^3^ for RV, 581.00–8460.33 for RT, 512.50–5852.80 for RF, 0.01–0.11 g for RDW, 0.04–0.61 g for SDW, 0.07–0.73 for DRS, 6.71–16.65 for RN, respectively (Table 1). Mean coefficients of variation (CV) was 28.62% for all traits (Table 1). Meanwhile, 2SY RILs showed bidirectionally transgressive segregation—phenotypic values beyond the values of parents—for all traits (Supplementary Figure 1 and Table 1). The *H*^2^ for the ten traits (TRL, RA, RD, RV, RT, RF, RDW, SDW, DRS, and RN) reached 71.69, 52.23, 85.33, 18.04, 51.03, 63.53, 67.58, 48.09, 41.32, and 40.62%, respectively (Table 1). The frequency distributions exhibited continuous variation with approximately normal distributions, indicating they were typical quantitative traits (Figure 2 and Supplementary Figure 1). Moreover, significant correlations (*P* < 0.05) were detected in most of the five independent tests for all traits in 2SY population (Supplementary Table 2).

**TABLE 1:** Phenotypic variation of RSA-related traits from five independent tests in 2SY population.

Trait (*H*^2^)	Test	Parents		RILs		
			
		20828	SY95-71	Range	Mean ± SD	CV (%)
TRL (cm)	T1	537.81*	424.71	239.17–1570.10	533.96 ± 221.39	41.46
(71.69%)	T2	528.15*	389.90	188.19–925.13	504.15 ± 151.42	30.04
	T3	595.99*	452.44	234.77–1931.78	908.54 ± 322.83	35.53
	T4	N	404.71	292.28–1465.78	685.11 ± 227.66	33.23
	T5	771.11*	595.52	324.35–1849.69	898.71 ± 275.18	30.62
	BLUP	637.96	527.91	455.43–993.63	705.87 ± 114.70	16.25
RA (mm^2^)	T1	60.16	53.27	26.35–195.94	66.30 ± 28.10	42.38
(52.23%)	T2	61.97*	48.26	21.19–125.28	61.63 ± 20.42	33.13
	T3	58.48	48.61	24.60–195.88	92.68 ± 32.95	35.55
	T4	N	47.78	33.32–148.14	74.67 ± 25.41	34.03
	T5	70.29*	55.41	34.69–189.93	90.72 ± 28.74	31.68
	BLUP	66.93	57.97	49.48–112.49	77.18 ± 13.34	17.28
RD (mm)	T1	0.36	0.40	0.33–0.47	0.39 ± 0.03	7.32
(85.33%)	T2	0.37	0.40	0.33–0.45	0.39 ± 0.03	6.79
	T3	0.31	0.34*	0.27–0.37	0.33 ± 0.02	6.81
	T4	N	0.39	0.28–0.42	0.35 ± 0.03	8.01
	T5	0.29	0.31	0.27–0.37	0.32 ± 0.02	6.26
	BLUP	0.35	0.36	0.34–0.37	0.35 ± 0.005	1.27
RV (mm^3^)	T1	0.53	0.54	0.23–1.95	0.66 ± 0.29	44.18
(18.04%)	T2	0.58	0.48	0.19–1.36	0.60 ± 0.23	37.41
	T3	0.46	0.42	0.21–1.58	0.76 ± 0.28	36.91
	T4	N	0.45	0.29–1.26	0.65 ± 0.24	36.47
	T5	0.51	0.42	0.30–1.66	0.73 ± 0.25	33.92
	BLUP	0.57	0.52	0.44–1.05	0.68 ± 0.12	18.10
RT (count)	T1	1633.50	1328.00	581.00–8460.33	1870.12 ± 1121.55	59.97
(51.03%)	T2	1985.67*	1311.33	647.25–3653.33	1528.42 ± 640.38	41.90
	T3	1292.80	1512.00	605.33–7347.50	3140.39 ± 1623.31	51.69
	T4	N	921.43	636.50–4759.67	2154.31 ± 872.02	40.48
	T5	3023.80*	1863.20	823.00–6029.33	2671.49 ± 883.34	33.07
	BLUP	2165.81	1943.72	1844.85–2892.60	2266.22 ± 198.99	8.78
RF (count)	T1	1696.25*	963.00	584.75–5501.67	1666.00 ± 798.73	47.94
(63.53%)	T2	1747.67**	893.00	512.50–3191.60	1556.95 ± 600.94	38.60
	T3	2147.60**	963.67	697.33–5852.80	2640.53 ± 1029.7	39.00
	T4	N	1341.43	738.13–5377.13	2018.68 ± 815.22	40.38
	T5	2975.20**	1683.80	840.29–5723.54	2747.66 ± 929.90	33.84
	BLUP	2117.56	1454.90	1429.83–3406.20	2125.13 ± 390.39	18.37
RDW (g)	T1	0.03	0.03	0.01–0.10	0.04 ± 0.02	43.63
(67.58%)	T2	0.02	0.02	0.01–0.07	0.03 ± 0.01	42.17
	T3	0.02	0.02	0.01–0.11	0.04 ± 0.01	36.12
	T4	N	0.03	0.01–0.07	0.03 ± 0.01	35.66
	T5	0.04	0.04	0.02–0.11	0.05 ± 0.02	30.93
	BLUP	0.03	0.03	0.03–0.05	0.04 ± 0.01	14.82
SDW (g)	T1	0.14	0.15	0.08–0.37	0.18 ± 0.07	35.85
(48.09%)	T2	0.13	0.15	0.04–0.44	0.20 ± 0.07	36.63
	T3	0.14	0.16*	0.08–0.45	0.24 ± 0.08	33.42
	T4	N	0.26	0.08–0.52	0.22 ± 0.10	44.27
	T5	0.17	0.28**	0.08–0.61	0.28 ± 0.09	31.68
	BLUP	0.17	0.21	0.16–0.32	0.23 ± 0.03	15.54
DRS	T1	0.19	0.20	0.10–0.73	0.21 ± 0.08	39.33
(41.32%)	T2	0.17	0.13	0.07–0.53	0.16 ± 0.06	38.12
	T3	0.17*	0.14	0.09–0.32	0.17 ± 0.05	27.28
	T4	N	0.12	0.07–0.27	0.15 ± 0.05	30.37
	T5	0.21	0.16	0.12–0.46	0.20 ± 0.04	22.08
	BLUP	0.18	0.16	0.13–0.25	0.18 ± 0.02	11.08
RN (count)	T1	11.00	9.50	7.00–15.00	9.97 ± 1.51	15.18
(40.62%)	T2	11.17*	9.67	8.50–15.40	11.21 ± 1.15	10.27
	T3	12.17	10.20	7.33–16.56	11.41 ± 1.57	13.77
	T4	N	9.71	7.44–14.11	10.56 ± 1.40	13.22
	T5	12.00**	9.56	6.71–15.75	11.36 ± 1.44	12.72
	BLUP	11.21	10.23	9.68–12.48	10.89 ± 0.48	4.38

*H*^2^ broad-sense heritability, *RIL* recombinant inbred line, SD standard deviation, *CV* coefficient of variation, *BLUP* best linear unbiased prediction, *TRL* total root length, *RA* root area, *RV* root volume, *RD* root diameter, *RT* root tips, *RF* root forks, *RDW* root dry weight, *SDW* shoot dry weight, *DRS* dry root–shoot ratio, *RN* root number; T represents test and N respresents the data was missed. * and ** mean significant difference at *P* < 0.05 and 0.01 probability level.

### Correlation analysis among root system architecture-related and agronomy traits

The BLUP datasets for RSA-related and agronomy traits were employed to assess their relationships. The Pearson’s correlations for RSA-related traits ranged from -0.34 to 0.99 (Figure 2). Significant and positive correlations (*P* < 0.05) were observed between RSA-related traits except RD and TRL, RT, and RF; DRS and RF; SDW and RN. However, DRS was negatively correlated with SDW (*P* < 0.01). Among them, the correlation coefficients between RA and TRL and RV were extremely high (reached 0.99) (Figure 2).

The relationships between RSA-related and agronomy traits were also estimated in Table 2. PH and SEL were significantly and positively correlated with TRL, RA, RV, RT, RF, RDW, and SDW (*P* < 0.05), and negatively correlated with DRS (*P* < 0.05). Significant and positive correlations were also detected between TN and RDW and SDW (*P* < 0.05); TGW and RN (*P* < 0.05). SNS was not significantly correlated with any RSA-related traits (Table 2).

**TABLE 2:** Correlation coefficients between RSA-related and agronomy traits.

	TRL	RA	RD	RV	RT	RF	RDW	SDW	DRS	RN
PH	0.34**	0.35**	0.06	0.35**	0.27**	0.33**	0.34**	0.53**	−0.22*	0.15
SEL	0.30**	0.29**	–0.04	0.28**	0.23*	0.26**	0.26**	0.54**	−0.32**	0.14
TN	0.14	0.15	0.13	0.16	0.15	0.04	0.20*	0.23**	–0.02	0.15
SNS	–0.01	0.01	0.14	0.02	–0.03	0.03	0.02	–0.11	0.17	–0.02
TGW	0.05	0.06	0.06	0.07	0.01	0.07	0.08	0.16	–0.11	0.20*

*TRL* total root length, *RA* root area, *RV* root volume, *RD* root diameter, *RT* root tips, *RF* root forks, *RDW* root dry weight, *SDW* shoot dry weight, *DRS* dry root–shoot ratio, *RN* root number, *PH* plant height, *SEL* spike extension length, *TN* tiller number, *SNS* spikelet number per spike, *TGW* thousand-grain weight. * and ** mean significant difference at *P* < 0.05 and 0.01 probability level.

### Quantitative trait loci mapping for root system architecture-related traits

A total of 28 putative QTL for 10 RSA-related traits covering thirteen chromosomal regions (chromosomes 1B, 2B, 2D, 3A, 4B, 4D, 5A, 5B, 5D, 6A, 6B, 7A, and 7B) were detected in five tests and BLUP datasets. Eleven positive alleles of these QTL were from 20828, and 17 were from SY95-71, individually explaining 6.66–38.48% of the phenotypic variations with a LOD value of 3.31–16.69 (Table 3).

**TABLE 3:** Quantitative trait loci (QTL) for RSA-related traits identified from 5 independent tests.

Traits	QTL	Test	Position (cM)	Flanking Markers	LOD value	PVE (%)	Add	Physical position (Mb)
TRL	*QTrl.sicau-2SY-2B*	T3	94	*AX-110580733–AX-109395848*	4.52	11.86	133.26	46.84–54.29
	*QTrl.sicau-2SY-4D*	T1	62	*AX-110527441–AX-110572006*	4.29	15.88	–93.58	14.96–19.59
		T2	61	*AX-110527441–AX-110572006*	8.47	25.19	–85.62	
		T3	63	*AX-110527441–AX-110572006*	6.65	15.81	–151.25	
		T4	63	*AX-110527441–AX-110572006*	4.58	15.97	–95.41	
		T5	60	*AX-110527441–AX-110572006*	8.17	26.03	–144.32	
		BLUP	62	*AX-110527441–AX-110572006*	14.62	38.48	–73.91	
RA	*QRa.sicau-2SY-2B.1*	T3	95	*AX-110580733–AX-109395848*	4.22	11.54	12.80	46.84–54.29
	*QRa.sicau-2SY-2B.2*	BLUP	82	*AX-111563183–AX-110442271*	3.76	8.36	3.82	45.78–45.73
	*QRa.sicau-2SY-4D*	T1	61	*AX-110527441–AX-110572006*	4.51	16.25	–12.44	14.96–19.59
		T2	61	*AX-110527441–AX-110572006*	6.50	22.60	–10.76	
		T3	62	*AX-110527441–AX-110572006*	6.88	18.39	–15.88	
		T4	63	*AX-110527441–AX-110572006*	4.52	15.33	–10.70	
		T5	61	*AX-110527441–AX-110572006*	7.50	24.12	–14.89	
		BLUP	62	*AX-110527441–AX-110572006*	12.51	34.70	–7.64	
RD	*QRd.sicau-2SY-6B*	BLUP	118	*AX-108816205–AX-111513561*	3.91	9.74	0.002	156.90–145.79
RV	*QRv.sicau-2SY-2B*	T3	95	*AX-110580733–AX-109395848*	4.53	12.50	0.11	46.84–54.29
	*QRv.sicau-2SY-4D*	T1	61	*AX-110527441–AX-110572006*	4.54	15.97	–0.13	14.96–19.59
		T2	61	*AX-110527441–AX-110572006*	5.36	19.34	–0.11	
		T3	62	*AX-110527441–AX-110572006*	6.75	17.35	–0.13	
		T4	62	*AX-110527441–AX-110572006*	3.94	13.45	–0.10	
		T5	61	*AX-110527441–AX-110572006*	6.30	20.75	–0.12	
		BLUP	62	*AX-110527441–AX-110572006*	12.55	34.54	–0.08	
RT	*QRt.sicau-2SY-1B*	T5	99	*AX-109308955–AX-110970287*	3.93	6.82	–276.51	477.39–476.56
	*QRt.sicau-2SY-3A*	T4	120	*AX-110645660–AX-110479868*	3.57	10.24	–307.51	606.20–628.15
	*QRt.sicau-2SY-4D*	T5	59	*AX-94547815–AX-110527441*	8.05	15.56	–416.61	15.42–14.96
		BLUP	63	*AX-110527441–AX-110572006*	6.98	22.10	–96.95	14.96–19.59
RF	*QRf.sicau-2SY-2D*	T2	9	*AX-110604633–AX-111526465*	4.53	10.38	204.75	70.44–69.86
	*QRf.sicau-2SY-4D.1*	T5	48	*AX-108735064–AX-94547815*	6.71	20.62	–393.74	12.27–15.42
	*QRf.sicau-2SY-4D.2*	T1	62	*AX-110527441–AX-110572006*	3.97	14.84	–326.61	14.96–19.59
		T2	62	*AX-110527441–AX-110572006*	7.18	19.18	–275.32	
		T3	62	*AX-110527441–AX-110572006*	5.23	18.26	–474.07	
		T4	63	*AX-110527441–AX-110572006*	4.31	14.69	–333.09	
		BLUP	62	*AX-110527441–AX-110572006*	12.41	32.68	–221.17	
	*QRf.sicau-2SY-6A*	BLUP	149	*AX-108876696–AX-109817601*	4.58	9.66	120.16	597.85–598.02
	*QRf.sicau-2SY-7B*	T5	163	*AX-108840818–AX-111586468*	3.79	10.74	283.78	581.75–575.44
RDW	*QRdw.sicau-2SY-4D*	T1	62	*AX-110527441–AX-110572006*	4.47	16.27	–0.01	14.96–19.59
		T5	61	*AX-110527441–AX-110572006*	5.18	15.60	–0.01	
		BLUP	62	*AX-110527441–AX-110572006*	9.10	26.02	–0.003	
SDW	*QSdw.sicau-2SY-4B.1*	BLUP	0	*AX-110928817–AX-111620391*	3.85	7.06	–0.01	38.72–40.61
	*QSdw.sicau-2SY-4B.2*	T3	3	*AX-111573292–AX-111233094*	4.60	17.22	–0.04	40.59–47.63
	*QSdw.sicau-2SY-4D*	T1	62	*AX-110527441–AX-110572006*	5.12	19.12	–0.03	14.96–19.59
		T5	63	*AX-110527441–AX-110572006*	8.03	16.07	–0.04	
		BLUP	63	*AX-110527441–AX-110572006*	12.38	27.80	–0.02	
	*QSdw.sicau*-*2SY-5B*	BLUP	0	*AX-108791526–AX-111490382*	4.04	7.43	–0.01	35.16–35.25
DRS	*QDrs.sicau-2SY-4B*	BLUP	52	*AX-109637078–AX-109861624*	7.12	6.66	0.01	519.23–529.15
	*QDrs.sicau*-*2SY-5A.1*	BLUP	17	*AX-109512380–AX-111572088*	8.11	7.72	–0.01	581.77–585.45
	*QDrs.sicau*-*2SY-5A.2*	T3	24	*AX-110373981–AX-110404585*	3.41	12.09	0.02	587.31–587.07
		BLUP	24	*AX-110373981–AX-110404585*	16.69	18.56	0.01	
	*QDrs.sicau*-*2SY-5D*	T4	40	*AX-108968976–AX-108881619*	4.12	15.12	0.02	54.93–59.50
	*QDrs.sicau*-*2SY-7A*	T3	41	*AX-110030320–AX-111499495*	3.31	11.75	–0.02	629.63–632.34
RN	*QRn.sicau*-*2SY-5B*	T5	14	*AX-110402737–AX-110586945*	3.74	13.28	–0.52	586.59–510.41
	*QRn.sicau-2SY-6B*	T4	6	*AX-89696866–AX-109900030*	3.88	13.50	–0.56	666.87–680.00

*LOD* logarithm of odds, *PVE* phenotype variance explained, *Add* additive effect of a QTL, *TRL* total root length, *RA* root area, *RV* root volume, *RD* root diameter, *RT* root tips, *RF* root forks, *RDW* root dry weight, *SDW* shoot dry weight, *DRS* dry root–shoot ratio, *RN* root number, *BLUP* best linear unbiased prediction; T represents Test; Positive value of Add indicates that alleles from 20828 are increasing the trait scores, and negative value of Add indicates that alleles from SY95-71 are increasing the trait scores; The physical position of each marker came from CS v2.1 genome.

Among them, six major QTL controlling TRL, RA, RV, RF, RDW, and SDW, were detected in at least two tests. *QTrl.sicau-2SY-4D*, *QRa.sicau-2SY-4D*, and *QRv.sicau-2SY-4D* can be stably expressed in all tests as well as BLUP datasets, explaining 15.81–38.48%, 15.33–34.70%, and 13.45–34.54% of phenotypic variation with a LOD value of 4.29–14.62, 4.51–12.51, and 3.94–12.55, respectively. *QRf.sicau-2SY-4D.2* was identified in four tests and with BLUP dataset except T5, explaining 14.69–32.68% of phenotypic variation with a LOD value of 3.97–12.41. *QRdw.sicau-2SY-4D* and *QSdw.sicau-2SY-4D* with a LOD value of 4.47–9.10 and 5.12–12.38 were detected in T1, T5, and BLUP dataset, accounting for 15.60–26.02% and 16.07–27.80% of the phenotypic variation, respectively. The remaining 22 minor QTL were unstable and detected in only one test, although they had a relatively high value of phenotypic variation (6.66–22.10%) (Table 3).

**FIGURE 3:**
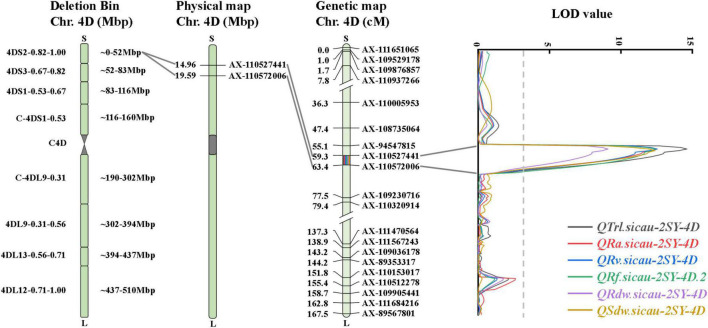
Genetic and physical maps of the major QTL.

Remarkably, there was a co-localized region for RSA-related traits. *QTrl.sicau-2SY-4D*, *QRa.sicau-2SY-4D*, *QRv.sicau-2SY-4D*, *QRf.sicau-2SY-4D.2*, *QRdw.sicau-2SY-4D*, and *QSdw.sicau-2SY-4D* were mapped between flanking markers *AX-110527441* and *AX-110572006* and located at a physical (genetic) interval of 14.96–19.59 Mb (59.3–63.4 cM) in the deletion bin 4DS2-0.82-1.00 on chromosome arm 4DS in CS v2.1 genome, and their positive alleles were from SY95-71 (Figure 3 and Table 3).

### Effects of the major QTL in HTG3SY, MZ5SY, and 2SY populations

**FIGURE 4:**
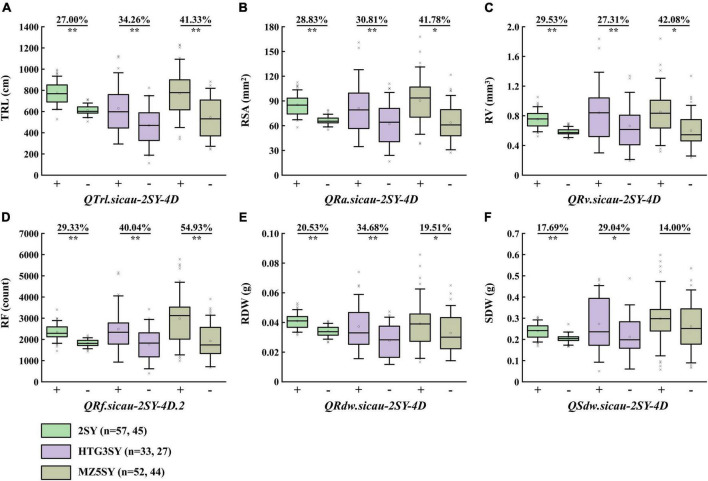
Effects of *QTrl.sicau-2SY-4D* **(A)**, *QRsa.sicau-2SY-4D* **(B)**, *QRv.sicau-2SY-4D* **(C)**, *QRf.sicau-2SY-4D.2* **(D)**, *QRdw.sicau-2SY-4D* **(E)**, and *QSdw.sicau-2SY-4D* **(F)** in 20828/SY95-71 (2SY), HTGW3/SY95-71 (HTG3SY), and MZ5-2/SY95-71 (MZ5SY) populations, respectively. + and - represent lines with and without the positive alleles of the corresponding QTL. * and ** mean significant difference at *P* < 0.05 and 0.01 probability level.

HTG3SY and MZ5SY populations were used to evaluate the effects of the co-localization QTL interval on corresponding traits in different genetic backgrounds (Figure 4). Its effect in 2SY population was also analyzed. *KASP-AX-110527441* tightly linked to the co-localization QTL interval was developed, and it can detect polymorphism between SY95-71 and HTGW3 and MZ5-2. Two categories with the homozygous alleles from HTGW3 or MZ-5 and SY95-71 were divided by *KASP-AX-110527441*. 2SY RILs were divided into two categories (carrying homozygous alleles either 20828 or SY95-71) by *AX-110527441* from Wheat55K SNP array (Liu et al., 2020). TRL, RA, RV, RF, RDW, and SDW of lines with homozygous alleles from SY95-71 got an immense increase (14.00–54.93%) in HTG3SY, MZ5SY, and 2SY population, respectively. For RDW, no significant difference was detected between the two categories in MZ5SY population (Figure 4).

Besides, effects of the co-localization QTL interval on other agronomic traits were evaluated in 2SY population as shown in Supplementary Figure 2. Lines carrying homozygous alleles from 20828 and SY95-71 were compared and the results showed that the co-localization QTL interval significantly affected (*P* < 0.05) the PH and SEL (Supplementary Figures 2A,B), whereas did not affect the TN, SNS, and TGW (Supplementary Figures 2C–E).

### Geographic distribution and effects of *QTrl.sicau-2SY-4D* in Chinese wheat landraces

**FIGURE 5:**
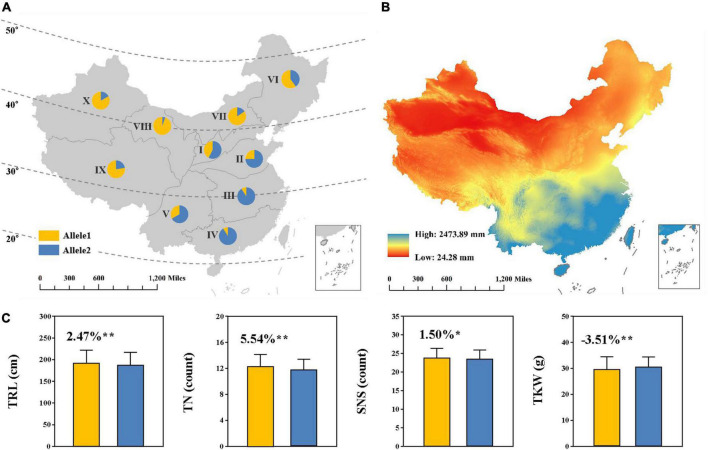
Geographic distribution **(A)** and effects **(C)** of *QTrl.sicau-2SY-4D* in Chinese wheat landraces (CWL) across 10 Chinese agro-ecological zones. Allele1 and Allele2 represent lines with and without the positive alleles of *QTrl.sicau-2SY-4D*, containing 207 and 369 lines, respectively. * and ** mean significant difference at *P* < 0.05 and 0.01 probability level. Lines with heterozygous alleles were excluded. The annual precipitation data during 2000–2015 in China **(B)** was provided by the Resource and Environment Science and Data Center, Institute of Geographic Sciences and Natural Resources Research (http://www.resdc.cn).

We further investigated the geographic distribution of the positive allele at *QTrl.sicau-2SY-4D* in wheat landraces across Chinese ten agro-ecological zones (Figure 5A). *KASP-AX-110527441* was used to determine the alleles of *QTrl.sicau-2SY-4D* in the panel of CWL concluding 717 wheat landraces (Supplementary Table 3). The result showed that the positive allele of *QTrl.sicau-2SY-4D* was present in all agro-ecological zones and its utilization degree varies greatly. The proportion of CWL carrying positive allele of *QTrl.sicau-2SY-4D* was 42.42, 25.20, 11.53, 9.09, and 33.33% in agro-ecological zones I, II, III, IV, and V, whereas reached 60.00, 84.62, 94.74, 77.61, and 83.33% in agro-ecological zones VI, VII, VIII, IX, and X, respectively. Interestingly, the mean annual precipitation of China during the period of 2000–2015 was inversely proportional to the utilization degree for the positive allele of *QTrl.sicau-2SY-4D* in Chinese ten agro-ecological zones (Figures 5A,B).

According to the genotypes of CWL, we further compared TRL, TN, SNS, and TGW between the lines with or without the positive allele at *QTrl.sicau-2SY-4D* (Figure 5C). Lines with positive alleles of *QTrl.sicau-2SY-4D* showed a significant increase (*P* < 0.05) in TRL, TN, and SNS by 2.47, 5.54, and 1.50%, but a significant decrease (-3.51%, *P* < 0.01) in TGW.

### Conditional quantitative trait loci analysis between total root length and other root system architecture-related traits, plant height, and spike extension length

**FIGURE 6:**
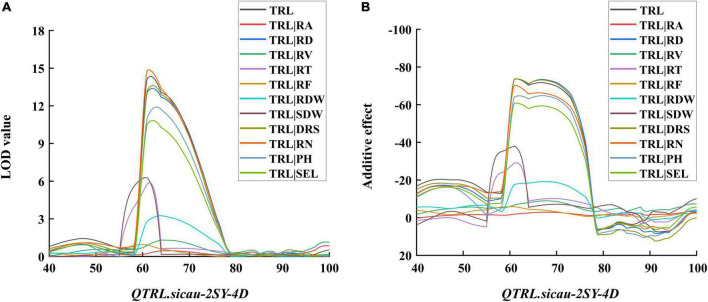
Logarithm of odds (LOD) **(A)** and additive effect **(B)** values of conditional QTL for total root length (TRL). *RA* root area, *RV* root volume, *RD* root diameter, *RT* root tips, *RF* root forks, *RDW* root dry weight, *SDW* shoot dry weight, *DRS* dry root–shoot ratio, *RN* root number, *PH* plant height, *SEL* spike extension length.

To further investigate the contribution of other RSA-related traits to TRL, the LOD and additive effect values of conditional QTL and unconditional QTL were compared (Figure 6 and Supplementary Table 4). The LOD value of *QTrl.sicau-2SY-4D* was significantly decreased when TRL was conditional on RA, RV, RT, RF, RDW, and SDW, but not significantly changed when it was conditional on RD, DRS, and RN (Figure 6A). The analysis of additive effect value was basically consistent with the LOD value (Figure 6B). The result suggested TRL was closely related to RV, RT, RF, RDW, and SDW, but independent of RD, DRS, and RN. The dwarfing genes were reported to have different effects on wheat root growth (Wojciechowski et al., 2009; Bai et al., 2013). Conditional QTL was thus carried out to evaluate the effect of PH and SEL on TRL. When TRL was conditional on PH and SEL, the LOD and additive effect values of *QTrl.sicau-2SY-4D* were slightly reduced, indicating the *QTrl.sicau-2SY-4D* was partially affected by PH and SEL (Figures 6A,B).

## Discussion

### The essentiality of detecting loci for root system architecture using seeds from different parental reproductive environments

The influence of PREs was reportedly to be carried over through the seeds to progenies (Roach and Wulff, 1987). PREs affected the seeds size, weight, and vigor of next generation which further impacting seedling germination and primary root growth (Elwell et al., 2011; Nosalewicz et al., 2016; Lorts et al., 2020). In this study, the seeds harvested from five different PREs showed different size as reported in our previous study (Qu et al., 2021). RSA for a given line from different PREs exhibited different phenotypes although under the same experimental conditions. These performances suggested PREs indeed significantly affects seeds and the germinated seedlings. This further affected the identification of QTL for RSA. For example, *QRn.sicau-2SY-5B* can be detected in only one test (i.e., PREs). Fortunately, a major locus, *QTrl.sicau-2SY-4D* for TRL was able to be detected in all of the five PREs. As far as we know, few of previous studies have considered the influence of PREs on the identification of QTL related to RSA and nearly no QTL RSA that were not affected by PREs were identified. Thus, this presently identified QTL, *QTrl.sicau-2SY-4D* should have great breeding value given it is likely independent of PREs.

### The positive allele of *QTrl.sicau-2SY-4D* was preferably selected in wheat breeding

Genetic analysis showed that the major QTL *QTrl.sicau-2SY-4D* was stably expressed in different PREs and its positive allele increasing root biomass in two parental populations (HTG3SY and MZ5SY) and one natural population (CWL) was validated (Figures 4, 5C). Moreover, we further found that utilization degree for the positive allele of *QTrl.sicau-2SY-4D* among CWL in northwest China where precipitation was significantly lower was significantly higher than that in southeast China (Figure 5A). This suggested that the positive allele of *QTrl.sicau-2SY-4D* has been preferably selected in wheat breeding in arid or semi-arid areas (Figures 5A,B). Plants may adopt different rooting strategies to meet changes in precipitation (Wang et al., 2020). Optimization of RSA allows crops to better capture water and nutrients and improve tolerance in extreme environments (Li et al., 2021; Maqbool et al., 2022).Therefore, we hypothesized that wheat growing in humid areas may not need a larger RSA to increase yield due to abundant precipitation, whereas in arid or semi-arid areas, wheat needs a better RSA to capture water and nutrients and satisfy the purpose of increasing yield. Thus, the local climate should be preferably considered when optimizing RSA in wheat breeding. Summarily, *QTrl.sicau-2SY-4D* is a major, stably expressed, and potentially valuable QTL. In short, *QTrl.sicau-2SY-4D* may be a locus related to precipitation distribution. Using the newly developed KASP marker (*KASP-AX-110527441*) for *QTrl.sicau-2SY-4D* associated with precipitation distribution, wheat lines with shorter and longer TRL can thus be selected for breeding utilization in ecotopes with different precipitation.

### Genetic relationships among root system architecture-related and agronomy traits

As a main trait for RSA, TRL is beneficial to increase yield potential and should be included in wheat ideotype (Maqbool et al., 2022). *QTrl.sicau-2SY-4D* showed pleiotropic effects on TRL, RA, RV, RF, RDW, and SDW (Figure 3 and Table 3). To further elucidate the genetic basis of TRL, we evaluated the genetic relationships between TRL and other RSA-related traits. Similar to previous studies (Ma et al., 2017; Fan et al., 2018; Li T. et al., 2020), TRL showed significantly positive correlation to RA, RV, RT, RF, RDW, SDW, DRS, and RN except RD (Figure 2). Further conditional analysis showed that the LOD score and additive effect of *QTrl.sicau-2SY-4D* changed significantly when TRL was conditional on RA, RT, RF, RDW, and SDW, which indicated that they contributed more to TRL than RD, DRS, RN (Figure 6).

Most traits of root and shoot were confirmed to be closely linked or pleiotropic by genetic analysis (Xie et al., 2017). The correlations between RSA-related and agronomy traits showed that RSA was closely related to morphology of shoot in wheat (Table 2 and Supplementary Figure 2). No major QTL for PH on 4D chromosome was detected (Liu et al., 2020), while *QSEL.sicau-2SY-4D.1* (Li C. et al., 2020) was detected and shared the same flanking marker (*AX-110572006*) as *QTrl.sicau-2SY-4D* in 2SY population. SEL also plays an important role in ideal plant type of wheat (Zhang et al., 2017). Conditional analysis further showed that *QTrl.sicau-2SY-4D* was partially affected by PH and SEL (Figure 6). *Rht-D1* had a significant effect on root growth at seedlings stage and may also affect SEL (Wojciechowski et al., 2009; Li C. et al., 2020). These results suggested that *Rht-D1* and *QTrl.sicau-2SY-4D* may interact to regulate wheat ideotype.

Unexpectedly, the effects of *QTrl.sicau-2SY-4D* on yield-related traits were not consistent under different backgrounds (Figure 5C and Supplementary Figure 2). This difference may be caused by the presence of other loci affecting *QTrl.sicau-2SY-4D* in CWL. Genetic architecture among yield-related traits is complicated due to the complex molecular mechanisms, polygenic nature, and influence of environment (Cao et al., 2020). The genetic basis of root contribution to yield is not fully clear, and further fine mapping and cloning work are needed to isolate candidate genes of *QTrl.sicau-2SY-4D* for functional analysis.

### Comparison with loci in previous studies

To further determine relationships between *QTrl.sicau-2SY-4D* and reported QTL, we compared their physical intervals on the CS v2.1 genome (Supplementary Table 5). Few QTL controlling TRL have been reported on the 4D chromosome. For instance, Kabir et al. (2015) identified a QTL *QTRL-4D* that was located between flanking markers *Xbarc1118* (9.19 Mbp) and *Rht-D1* (19.19 Mbp) and further confirmed that it was closer to *Xbarc1118* in the linkage map. Bai et al. (2013) also identified a QTL *qTRL-4D* on 4D chromosome (127.17 Mbp). Both of them were unlikely allelic to *QTrl.sicau-2SY-4D*.

*Rht-D1* was found in the physical interval of *QTrl.sicau-2SY-4D* (14.96–19.59 Mbp) (Supplementary Table 6). The functional marker *KASP-Rht-D1* (Rasheed et al., 2016) for *Rht-D1* was used to genotype 2SY population and remapping QTL for TRL showed that *QTrl.sicau-2SY-4D* was not linked to *Rht-D1* (Supplementary Figure 3A,B). In view of the fact that *Rht-D1* had a significant influence on TRL (Supplementary Figure 3C) and *QTrl.sicau-2SY-4D* was partially affected by PH in 2SY population (Figure 6), further investigations are required to clarify the relationship between *Rht-D1* and *QTrl.sicau-2SY-4D*.

### Prediction of candidate genes associated with root system architecture

We found 96 high-confidence genes in the physical interval of *QTrl.sicau-2SY-4D* on CS genome, 75 genes of them were common predicated genes between CS v2.1 and Aet v5.0 genomes (Supplementary Table 6). Further expression analyses showed that 67 genes of the predicated common genes were expressed in roots (Supplementary Table 6). Four genes existed significantly higher expression levels in roots than other tissues and may be associated with RSA (Supplementary Figure 4). The change of RSA is an effective way for crops to obtain an efficient P acquisition ability (Niu et al., 2013). *TraesCS4D03G0059800* encodes a haloacid dehalogenase-like hydrolase, and it has a homologous gene (*OsHAD1*) that enhanced phosphate accumulation in rice (Pandey et al., 2017). *TraesCS4D03G0057800* encodes a pescadillo homolog which was reported to play an important role in plant cell growth and root development in *Arabidopsis* (Zografidis et al., 2014). Additionally, *TraesCS4D03G0064000* (a homolog of *eRF1-1*, *eRF1-2*, and *eRF1-3*) encodes a eukaryotic peptide chain release factor subunit 1-1 which impacted early seedling development in *Arabidopsis* (Zhou et al., 2010). *TraesCS4D03G0064400* encodes a Zinc finger family protein and potentially affects root formation (Cui et al., 2017). Collectively, these predicated genes associated with RSA may provide pivotal information for cloning and utilization of the *QTrl.sicau-2SY-4D*.

## Conclusion

We identified twenty-five putative QTL for RSA-related traits, covering twelve chromosomal regions. A major QTL, *QTrl.sicau-2SY-4D* for total root length which was likely independent of PREs, located at 14.96–19.59 Mb on chromosome arm 4DS. It showed pleiotropic effects on TRL, RA, RV, RF, RDW, and SDW. The KASP marker linked to this major QTL, was developed and used to successfully validate its effect in three different genetic populations. We further analyzed and discussed the contribution and geographical distribution of this major QTL. The major genetic locus of TRL and tightly linked KASP marker will be useful in wheat breeding and further gene cloning.

## Data availability statement

The original contributions presented in this study are included in the article/Supplementary material, further inquiries can be directed to the corresponding author.

## Author contributions

HC finished the study and wrote the manuscript. JW participated in glasshouse work, phenotype measurement, and data analysis. RT and ZZ helped phenotype measurement. HT and YLL helped data analysis and makers development. QX and MD collected and analyzed data. QJ, GC, YXL, WL, and PQ helped with data analysis and revision. YFJ, YJ, and LT performed marker analysis. YW revised the manuscript. YZ and XL discussed the results. JM designed the experiments, guided the entire study, participated in data analysis, and extensively revised this manuscript. All authors participated in the study and approved the final manuscript.

## Footnotes

1
https://www.epson.com.cn/

2
https://www.regentinstruments.com/

3
http://www.resdc.cn

4
https://www.sas.com

5
http://en.wikipedia.org/wiki/SPSS

6
https://www.originlab.com/

7
https://www.esri.com/

8
https://www.wur.nl/en/show/Mapchart.htm

9
https://wheat.triticeaetoolbox.org/

10
https://graingenes.org/GG3

## Supplementary material

Supplementary Figure 1 Frequency distribution of RSA-related traits for five tests and with BLUP datasets. *TRL* total root length **(A)**, *RA* root area **(B)**, *RV* root volume **(C)**, *RD* root diameter **(D)**, *RT* root tips **(E)**, *RF* root forks **(F)**, *RDW* root dry weight **(G)**, *SDW* shoot dry weight **(H)**, *DRS* dry root–shoot ratio **(I)**, *RN* root number **(J)**.

Supplementary Figure 2 Effects of *QTrl.sicau-2SY-4D* on other agronomic traits in 20828/SY95-71 (2SY) population. + and - represent lines with and without the positive alleles of *QTrl.sicau-2SY-4D*. *PH* plant height **(A)**, *SEL* spike extension length **(B)**, *TN* tiller number **(C)**, *SNS* spikelet number per spike **(D)**, TGW thousand-grain weight **(E)**. * and ^**^ mean significant difference at *P* < 0.05 and 0.01 probability level.

Supplementary Figure 3 Reintegration of genetic map for 2SY population with the inclusion of *KASP-Rht-D1* **(A,B)** and effect of *Rht-D1* on total root length (TRL) **(C)**. Partial genotypes were showed in Supplementary Figure 3. Blue box and orange frame represent lines with and without the positive allele of *Rht-D1* (HEX fluorescence and FAM fluorescence or + and −), green triangle represents lines with heterozygous alleles. ^**^Means significant difference at 0.01 probability level.

Supplementary Figure 4 Expression patterns of *TraesCS4D03G0059800* **(A)**, *TraesCS4D03G0057800* **(B)**, *TraesCS4D03G0064000* **(C)**, and *TraesCS4D03G0064400* **(D)** in different tissues of wheat.

Supplementary Table 1 Information of kompetitive allele-specific PCR (KASP) used in this study.

Supplementary Table 2 Correlation coefficients of five independent tests for root system architecture(RSA)-related traits.

Supplementary Table 3 Genotypes of Chinese wheat landraces (CWL).

Supplementary Table 4 Quantitative trait loci (QTL) detected for total root length (TRL) using conditional QTL analysis method.

Supplementary Table 5 Comparison of the major quantitative trait loci (QTL) for total root length (TRL) identified in this study with previous studies.

Supplementary Table 6 The annotation and expression in root for genes in the interval of *QTrl.sicau-2SY-4D*.
