# Supplementary figures and images for: A major quantitative trait locus for wheat total root length associated with precipitation distribution
Source: Front Plant Sci. 2022 Aug 24;13:995183. doi: 10.3389/fpls.2022.995183 (PMC9451531; doi:10.3389/fpls.2022.995183)

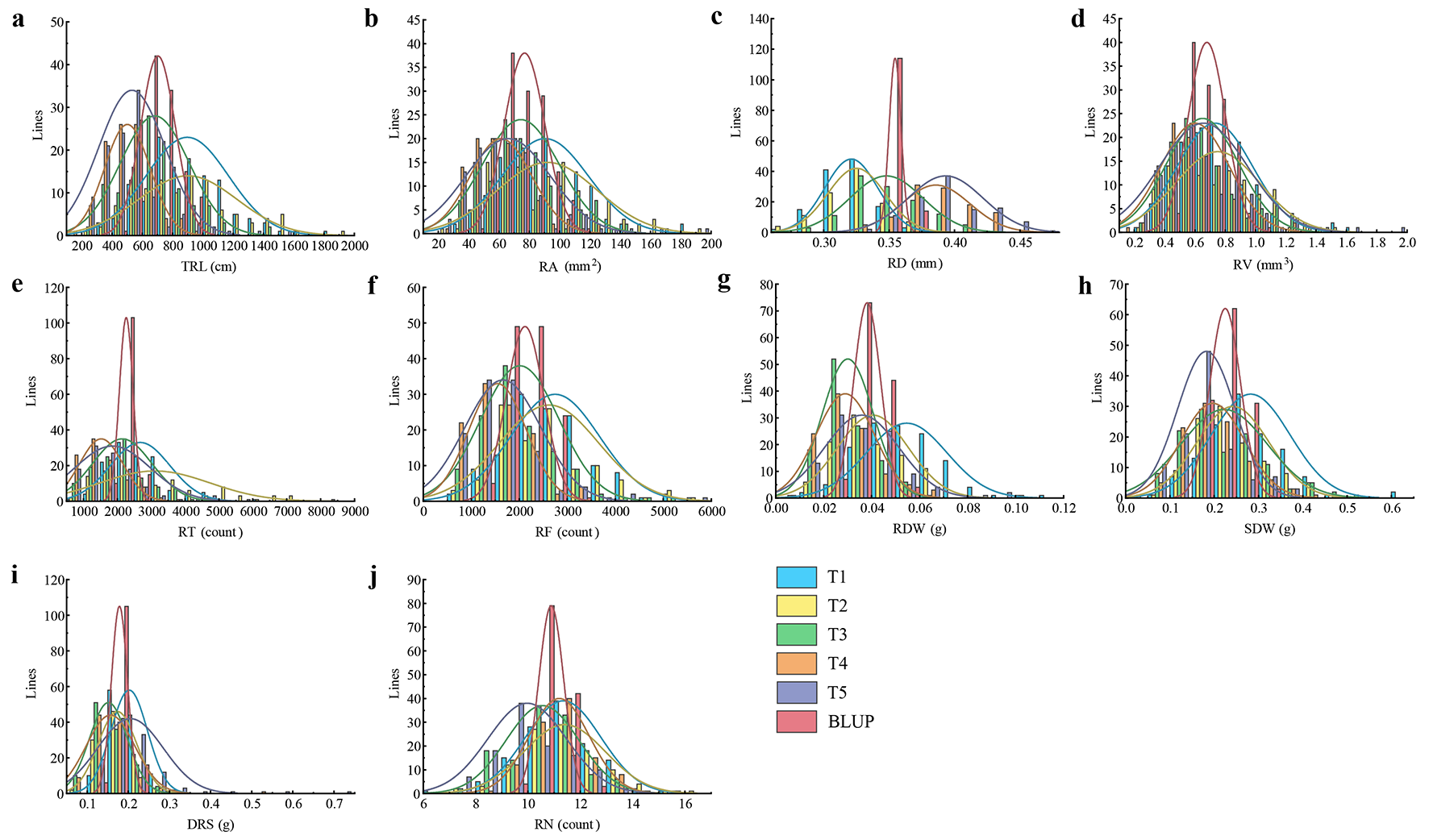

Supplement: Supplementary Figure 1 — Frequency distribution of RSA-related traits for five tests and with BLUP datasets. TRL total root length (A), RA root area (B), RV root volume (C), RD root diameter (D), RT root tips (E), RF root forks (F), RDW root dry weight (G), SDW shoot dry weight (H), DRS dry root–shoot ratio (I), RN root number (J). [file Image_1.TIF]

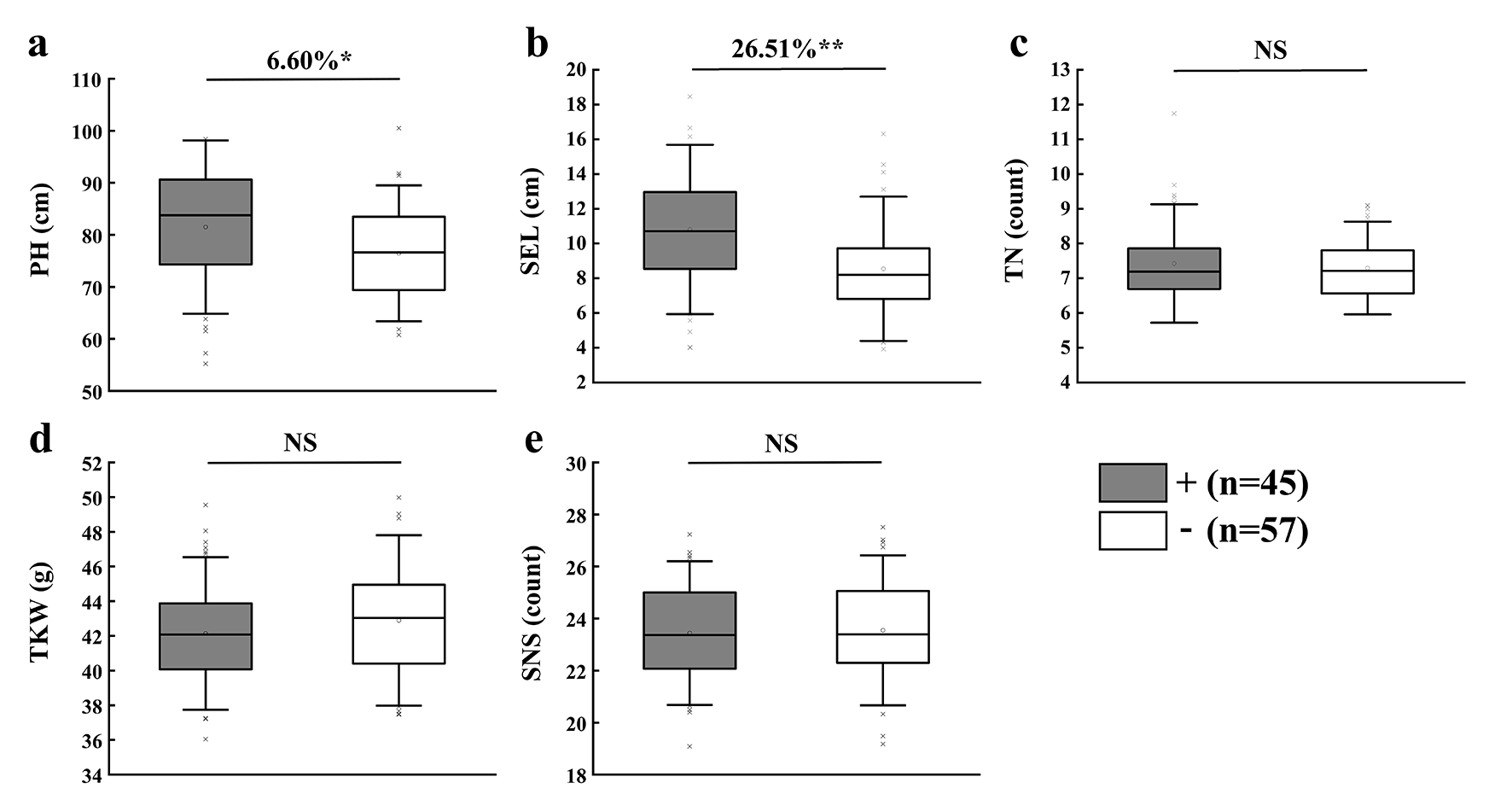

Supplement: Supplementary Figure 2 — Effects of QTrl.sicau-2SY-4D on other agronomic traits in 20828/SY95-71 (2SY) population. + and - represent lines with and without the positive alleles of QTrl.sicau-2SY-4D. PH plant height (A), SEL spike extension length (B), TN tiller number (C), SNS spikelet number per spike (D), TGW thousand-grain weight (E). * and ** mean significant difference at P < 0.05 and 0.01 probability level. [file Image_2.TIF]

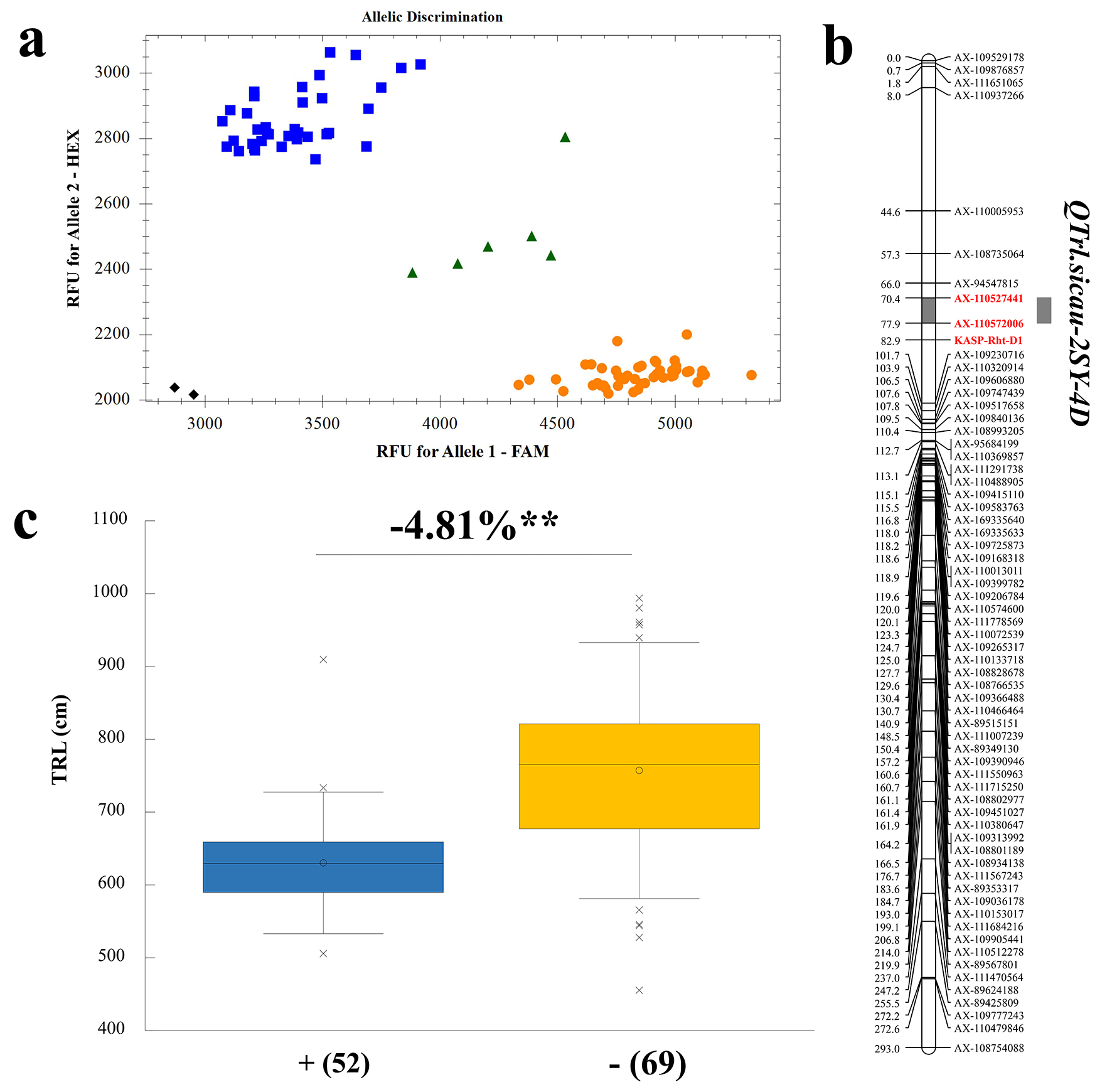

Supplement: Supplementary Figure 3 — Reintegration of genetic map for 2SY population with the inclusion of KASP-Rht-D1 (A,B) and effect of Rht-D1 on total root length (TRL) (C). Partial genotypes were showed in Supplementary Figure 3. Blue box and orange frame represent lines with and without the positive allele of Rht-D1 (HEX fluorescence and FAM fluorescence or + and −), green triangle represents lines with heterozygous alleles. **Means significant difference at 0.01 probability level. [file Image_3.TIF]

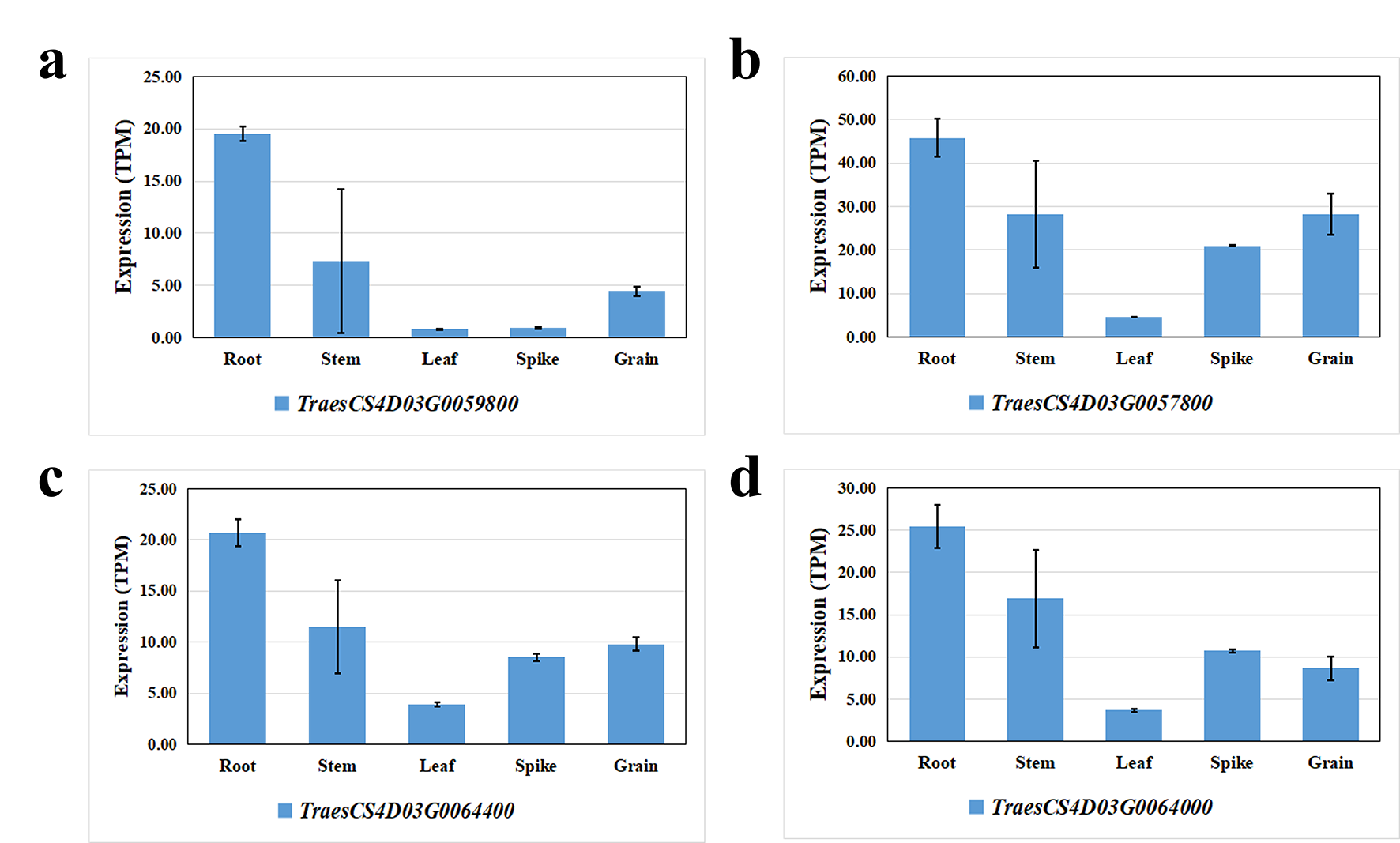

Supplement: Supplementary Figure 4 — Expression patterns of TraesCS4D03G0059800 (A), TraesCS4D03G0057800 (B), TraesCS4D03G0064000 (C), and TraesCS4D03G0064400 (D) in different tissues of wheat. [file Image_4.TIF]
